# Disassembly and reassembly of human papillomavirus virus-like particles produces more virion-like antibody reactivity

**DOI:** 10.1186/1743-422X-9-52

**Published:** 2012-02-22

**Authors:** Qinjian Zhao, Yorgo Modis, Katrina High, Victoria Towne, Yuan Meng, Yang Wang, Jaime Alexandroff, Martha Brown, Bridget Carragher, Clinton S Potter, Dicky Abraham, Dave Wohlpart, Mike Kosinski, Mike W Washabaugh, Robert D Sitrin

**Affiliations:** 1 Merck Research Laboratories, West Point, PA 19486, USA; 2 Department of Molecular Biophysics and Biochemistry, Yale University, 266 Whitney Ave, New Haven 06520, CT, USA; 3 Nano Imaging Services, 10931 North Torrey Pines Road, Suite 108, San Diego, CA 92037, USA; 4 Vaccine Manufacturing Science and Commercialization, Merck Manufacturing Division, West Point, PA 19486, USA; 5 School of Public Health, Xiamen University, Xiamen, Fujian 361005, China; 6 Yale University, 266 Whitney Ave, bass 430, New Haven, CT 06520, USA; 7 Sino Biologicals, Inc, Beijing, China; 8 MedImmune, Gaithersburg, MD 20878, USA

**Keywords:** Recombinant subunit vaccine, Virus-like particle (VLP), Neutralizing monoclonal antibody, Redox treatment, Competitive fluorescence ELISA, Epitope mapping, Atomic homology model

## Abstract

**Background:**

Human papillomavirus (HPV) vaccines based on major capsid protein L1 are licensed in over 100 countries to prevent HPV infections. The yeast-derived recombinant quadrivalent HPV L1 vaccine, GARDASIL(R), has played an important role in reducing cancer and genital warts since its introduction in 2006. The L1 proteins self-assemble into virus-like particles (VLPs).

**Results:**

VLPs were subjected to post-purification disassembly and reassembly (D/R) treatment during bioprocessing to improve VLP immunoreactivity and stability. The post-D/R HPV16 VLPs and their complex with H16.V5 neutralizing antibody Fab fragments were visualized by cryo electron microscopy, showing VLPs densely decorated with antibody. Along with structural improvements, post-D/R VLPs showed markedly higher antigenicity to conformational and neutralizing monoclonal antibodies (mAbs) H16.V5, H16.E70 and H263.A2, whereas binding to mAbs recognizing linear epitopes (H16.J4, H16.O7, and H16.H5) was greatly reduced.

Strikingly, post-D/R VLPs showed no detectable binding to H16.H5, indicating that the H16.H5 epitope is not accessible in fully assembled VLPs. An atomic homology model of the entire

HPV16 VLP was generated based on previously determined high-resolution structures of bovine papillomavirus and HPV16 L1 pentameric capsomeres.

**Conclusions:**

D/R treatment of HPV16 L1 VLPs produces more homogeneous VLPs with more virion-like antibody reactivity. These effects can be attributed to a combination of more complete and regular assembly of the VLPs, better folding of L1, reduced non-specific disulfide-mediated aggregation and increased stability of the VLPs. Markedly different antigenicity of HPV16 VLPs was observed upon D/R treatment with a panel of monoclonal antibodies targeting neutralization sensitive epitopes. Multiple epitope-specific assays with a panel of mAbs with different properties and epitopes are required to gain a better understanding of the immunochemical properties of VLPs and to correlate the observed changes at the molecular level. Mapping of known antibody epitopes to the homology model explains the changes in antibody reactivity upon D/R. In particular, the H16.H5 epitope is partially occluded by intercapsomeric interactions involving the L1 C-terminal arm. The homology model allows a more precise mapping of antibody epitopes. This work provides a better understanding of VLPs in current vaccines and could guide the design of improved vaccines or therapeutics.

## Background

The use of recombinant virus-like particles (VLP) as immunogens or vaccines has proven increasingly successful in recent years [1]. Most vaccines against viral diseases have traditionally relied on attenuated virus strains or inactivation of infectious virus. Self-assembly of recombinant viral capsid proteins and corresponding capsomeres into empty capsids is a promising strategy for production and design of virus-like particles (VLPs) for contemporary vaccines. The resulting VLPs may elicit a protective immune response by mimicking the authentic epitopes of virions. Recent VLP-based HPV vaccines (quadrivalent GARDASIL^® ^from yeast and bivalent Cervarix^® ^from insect cells) have been successful in preventing HPV infection and- HPV-related cancer-associated genital warts [2-7]. HPV virions contain 360 copies of L1 and up to 72 copies of L2, which assemble into an icosahedral, T = 7 structure of 55-60 nm in diameter with one L2 molecule being at the central opening of each capsomere [8]. L1 alone, when expressed in insect or yeast cells, self-assembles into VLPs. The VLP stability can be improved by oxidative maturation [9,10] or reassembly [11,12]. The immunogenicity of purified VLPs that did not undergo a reassembly step was confirmed through preclinical and early clinical studies using HPV 16 L1-derived VLPs expressed in yeast (*Saccharomyces cerevisiae*).

The spontaneous organization inside yeast cells of pentameric L1 capsomeres into periodically packed quasi-symmetric VLPs is controlled by thermodynamic constraints via the combination of many intra- and intercapsomeric forces. However, heterogeneity due to assembly polymorphism is common for VLPs lacking genetic material [13-15], in addition to a certain degree of aggregation and formation of incomplete capsids during expression in yeast cells and downstream bioprocessing. During the production of HPV16 L1 VLPs, disassembly and reassembly (D/R) treatment was employed during the bioprocessing to further improve the VLP immunoreactivity, homogeneity and stability. Disassembly was achieved with high pH, low salt and presence of reducing agent to harness the presumed intrinsic conformation switching mechanism of disassembly into capsomeres during the viral entry and endoplasmic uncoating of the HPV virions [12,16]. Removal of these disassembling agents under controlled conditions enabled consistent particle reassembly from capsomeres, yielding more homogeneous and fully assembled VLPs. The characterization of the reassembled HPV 16 VLPs was previously reported [12,16].

Here we quantify the impact of the D/R process on epitope specific antigenicity of full-scale production lots of HPV16 VLPs. Implementation of the D/R treatment during bioprocessing was one of the crucial steps in assuring the stability of VLPs, during pivotal clinical trials and eventually in Gardasil^® ^(licensed for human use in 2006). A panel of non-overlapping mAbs (Table 1), recognizing conformational as well as linear epitopes, was utilized as orthogonal molecular probes for the surface structural and functional assessment of the reassembled HPV 16 VLPs. Using solution competitive enzyme-linked immunosorbent assay (ELISA), the binding to the VLPs for three neutralizing Abs showed several fold higher antibody binding in the clinically relevant epitopes. In contrast, three non- or weakly-neutralizing mAbs recognizing conserved linear epitopes, showed a significant decrease in antibody binding to reassembled VLPs. In addition, the particle morphology and binding of the highly neutralizing H16.V5 Fab was visualized directly by cryo electron microscopy. Based on previously determined high-resolution structures of bovine papillomavirus and HPV16 L1 pentameric capsomeres, we generated an atomic homology model of the entire HPV16 VLP. Mapping of known antibody epitopes to the homology model explains the changes in antibody reactivity to the more virion-like fully closed VLPs upon reassembly.

**Table 1:** Characteristics of the HPV 16 mAbs used in the epitope specific antigenicity analysis.

mAb (subclass)	Neutralizing activity	Type specificity	Epitope [critical amino acids] (Refs)	*K_D _*(nM)^c^	Application^d^
*Conformational epitopes*					
H16.V5 (G2b)	Strong (330)^b^	Yes	FG loop, residues 266-297 (and HI loop, residues 339-365), [282, 50] [17,18]	< 0.1	cLIA, IVRP, IC50 [19-22]
H16.E70 (G2b)	Strong (1)^b^	Yes	Similar to V5, [285, 288, 266, 282] [23,24]	0.15	IC50
H236.A2	Strong (11)^b^	Yes	HI loop, residues 339-365 (and FG loop 266-297)	< 0.1	IC50
*Linear epitopes*^e^					
H16.J4 (G2a)	Weak	No	261-280 [17,25]	2-5	IVRP, IC50 [19,20]
H16.O7	No	No	174-185	~2	
H16.H5 (G2b)	No	No	174-185 [23]		

H16.V5 was identified as the mAb with highest affinity and also with highest neutralization efficiency a

^a ^Due to the immunodominant nature of this epitope in the sera of naturally infected individuals [26] and its high affinity to VLPs, V5 is the detection Ab for the sandwich ELISA for product batch release and stability testing [19,20]. In addition, it is also the mAb for the clinical serological assay with V5 as the marker in the competition RIA, ELISA or Luminex based assay for analyzing the sera from vaccines during clinical trials or post-licensure monitoring [21,22]

^b ^Relative neutralization efficiency (value in parenthesis) for the mAb was normalized to E70 [27]

^c ^The K_D _values are estimated with equilibrium based, indirect binding ELISA or SPR based methods with post-D/R VLPs [28,29]

^d ^In support of Gardasil^® ^development and commercial manufacturing

^e ^Most linear epitopes are not type specific. The linear epitopes, such as that of J4, were identified with immunoreactivity of synthetic peptides to the sera of patients with HPV infection and cervical neoplasms [25,30]

## Results

### Effects of reassembly on individual epitopes by solution antigenicity analysis

**Figure 1:**
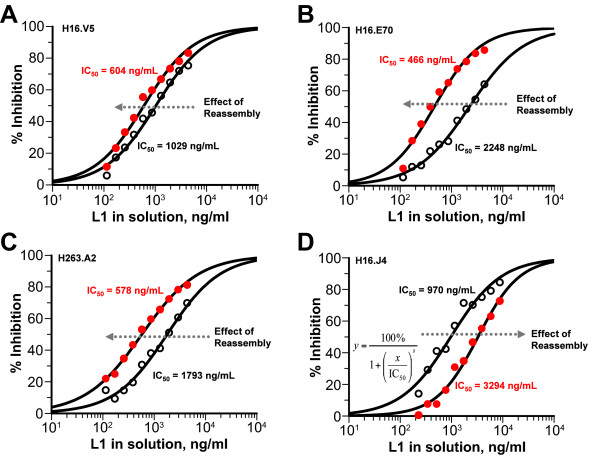
**Competitive fluorescence ELISA curves for H16.V5 (**A**), H16.E70 (**B**), H263.A2 (**C**) (conformational epitope and neutralizing) and H16.J4 (**D**) (linear epitope and weakly neutralizing) on a pair of pre- and post-D/R HPV 16 L1 VLP lots**. Fitted curves and the IC50 values were obtained with GraFit (20). Open dots correspond to pre-D/R VLPs; closed dots correspond to post-D/R VLPs. The relative IC50 values (normalized to a reference lot) for multiple full-scale lots are listed in Table 2.

Studies were initiated to better understand the impacts of the D/R process on the presumed clinically relevant epitopes-particularly those known to be neutralizing in pseudovirion neutralization assay [17,18,27] and to be immunodominant when analyzed by competition assay using human sera of naturally infected individuals [26]. Epitope specific antigenicity is important in understanding of the VLP structures, particularly the presence of the neutralizing epitopes and resemblance to authentic virions. The properties and characteristics of the six mAbs used in a solution antigenicity assessment by a competitive fluorescence ELISA method are listed in Table 1. H16.V5 is the best-characterized mAb against HPV 16 and has the highest neutralizing efficiency and activity of all mAbs studied [27,31]. H16.V5 recognizes an epitope which is made of two peptide stretches coming from two different loops of the L1 molecule-the FG loop (266-297) and secondarily the HI loop (339-365) of the adjacent L1 in the pentamer [27,32]. H16.V5 is used as the detection Ab in a sandwich ELISA for VLP lot release and product stability [19,20]. In order to dissect the impact on individual epitopes on the VLPs due to D/R, a solution competitive ELISA was developed, using one mAb at a time. The format was based on equilibrium dissociation constant determination [28,33] except that there was no need to reach equilibrium and no need to transfer a premixed Ag-Ab solution to the Ag coated plate (see Methods). Solution antigenicity was determined with VLPs in solution (both test sample and reference), binding to a fixed amount of mAb in solution, in competition to a pre- or post-D/R "reagent" VLP sample immobilized passively on the surface of the plate. Figure 1 illustrates the opposing effects on the mAb binding behaviors of VLPs pre- and post-D/R treatment for H16.V5, H16.E70, H236.A2 and H16.J4 with the inhibition profiles from the competitive ELISA. Quantitation of the relative antigenicity was achieved by comparing the IC50 values of test samples with that of a reference lot. Specifically in Figure 1, a pair of pre- and post-D/R VLP samples from the same production batch were used for comparative analysis. Not surprisingly, after breaking up some aggregates in the purified VLPs, the conformational and neutralizing Ab binding was increased by a factor of 2-3.5-fold, as reflected by a decrease in IC50 values as a result of disassembly (Figure 1A-C). The impact of D/R on the solution antigenicity is indicated by the arrow in Figure 1, with left movement indicating enhanced antibody binding in solution for post-reassembly VLPs. This is the desirable outcome of the D/R process for yielding VLPs with optimized H16.V5 epitope quality and quantity due to the immunodominant nature and high neutralizing efficiency of H16.V5. The enhanced V5 binding is an indication of more virion-like VLPs as a result of D/R. In contrast, the H16.J4 binding was reduced by ~5-fold upon VLP reassembly (Figure 1D). The D/R process was also highly efficient and consistent with a yield greater than 75%.

### Consistency of the D/R process

**Figure 2:**
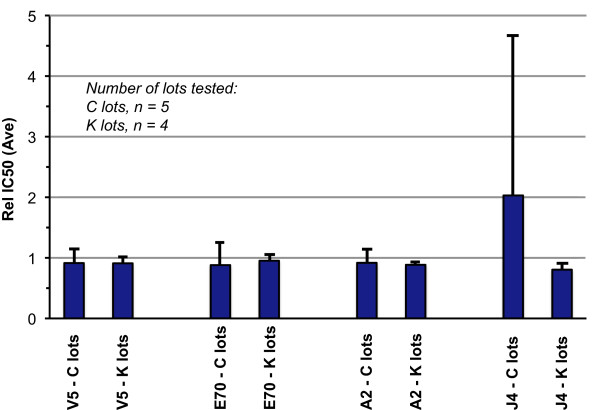
**Consistency in epitope specific antigenicity for clinical stage developmental lots ("C" lots) and commercial lots ("K" lots) as probed with a panel of mAbs**. Four mAbs were employed to track the solution antigenicity of the various lots (see relative IC50 values in Table 2) to provide additional quantitative information on VLP epitopes, in addition to product release tests. The one standard deviations were plotted for clinical lots (n = 5) and for commercial lots (n = 4) to illustrate the tighter range of VLP solution antigenicity of commercial lots achieved as compared to clinical developmental lots.

Interestingly, the D/R process, similar to viral uncoating and virion assembly inside the cell, could be performed with recombinant L1 reliably with high yield and consistency. Neutralizing epitopes on L1 VLPs were improved as desired, whereas a decrease of binding was observed for mAbs targeting linear epitopes as a result of D/R. A set of epitope-specific antigenicity data from IC50 assays with four mAbs is presented in Table 2. During the developmental stages of Gardasil^®^, five lots were manufactured during the clinical testing stage with one lot (C1) being just the purified VLPs (without D/R). High consistency was demonstrated for the VLP D/R process at production scale when multiple lots of post-D/R VLPs were analyzed. Figure 2 shows the epitope-specific antigenicity for each mAb as defined by the five developmental lots in clinical testing stage ("clinical experience", as specified in Table 2), and the actual process performance during post licensure production for commercial lots or "manufacturing experience". Consistency was demonstrated with respect to the D/R process in improving the clinically relevant epitopes on VLPs during late development phase and post licensure vaccine production. As indicated from the range of the commercial lots, we can conclude that a) the D/R process was successfully implemented at full scale; b) the range for the commercial lots is tighter than the lots for gaining "clinical experience" (due to the one pre-D/R lot used in early clinical trials); c) the multifaceted epitope-specific antigenicity assessment (Table 2 and Figure 2) is critical to assure the product comparability during a production scale-up or a process upgrade.

**Table 2:** Manufacturing consistency and epitope specific antigenicity testing of multiple lots of HPV16 VLPs-pre- and post-D/R treatment.

Lot No.^a^	Epitope specific antigenicity (Relative IC50)			
	**H16.V5**	**H16.E70**	**H263.A2**	**H16.J4**
*pre-D/R*				
C1	0.54	0.30	0.53	6.73
C2(pre-)	0.51	0.31	0.51	5.51
C3(pre-)	0.48	0.27	0.46	5.24
***Ave. (n = 3)***	***0.51 ± 0.03***	***0.29 ± 0.02***	***0.50 ± 0.03***	***5.83 ± 0.80***
*post-D/R*				
C2	1.17	1.33	1.11	0.43
C3	0.89	0.94	0.95	1.05
C4	0.97	0.83	1.00	0.94
C5^b^	1.00	1.00	1.00	1.00
K1	1.01	1.08	0.88	0.91
K2	0.76	0.83	0.82	0.73
K3	0.93	0.95	0.92	0.70
K4	0.94	0.95	0.92	0.88
***Ave. (n = 8)***	***0.96 ± 0.10***	***0.99 ± 0.07***	***0.95 ± 0.06***	***0.83 ± 0.10***

Solution antigenicity was assessed with a panel of four anti-HPV 16 L1 mAbs in a competitive FL-ELISA. Data from three lots of pre-D/R and eight lots of post-D/R are presented. Each reported relative IC50 value is an averaged value from three independent measurements on three different plates with a typical RSD% of 12-15%

^a ^Developmental lots during clinical testing stage ("C lots") and commercial lots ("K Lots")-see also Figure 2. Once the reassembly process was developed, only the post-D/R lots were tested clinically. Therefore, no clinical experience was gained with C2-pre-D/R and C3-pre-D/R, and these two lots are excluded from the analysis and plotting for "clinical experience" (Figure 2)

^b ^This lot was used as the reference lot in the relative antigenicity assay (or relative IC50). Lot C5 was used as the reference lot for all antigenicity testing and it was tested on every plate along with other test lots

### Quantitating the changes in different epitopes upon VLP reassembly

**Figure 3:**
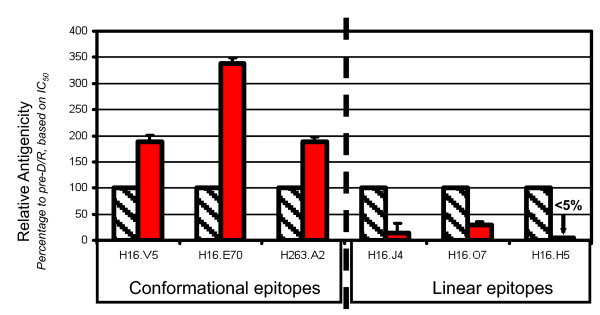
**Quantitative assessment of the impact of D/R on the reactivities of different antibodies to HPV16 VLPs by ELISA**. Bar graph of relative IC50 values (after normalizing to the pre-D/R control, based on the data in Table 2) for a panel of mAbs against post-D/R VLPs (solid bars) as compared to pre-D/R VLPs (hashed bars). A larger value indicates higher antigenicity based on the half-maximal binding titers-initially derived in ng/mL for L1 concentration in VLP preparations.

The impact on the antigenicity of VLPs probed by different mAbs is shown in Figure 3. Significant improvements in the IC50, ~2-3.5-fold, were observed for the epitopes for the conformation sensitive and neutralizing mAbs-H16.V5, H16.E70 and H263.A2. Conversely, the binding of three mAbs with linear epitope was markedly reduced (Figure 3).

It was particularly striking that the epitope for H5 was no longer detectable upon D/R treatment (Figure 3). In the direct Ag binding experiments, H5 showed significant binding to pre-D/R VLPs, but not to post-D/R VLPs. This was also confirmed with the solution competition ELISA or IC50 assays. There was no detectable H5 binding to post-D/R VLPs even in the solution competition format where the VLPs are allowed to freely interact with detecting mAb (H5) with only unbound H5 being detected by surface immobilized VLPs. The reduction of antibody reactivity with these linear epitopes and even the disappearance of reactivity with H16.H5 upon D/R is consistent with results from a recent report in which three mAbs (H5 in particular) targeting linear epitopes study were shown to have little or no binding to pseudovirions (structurally more similar to virions as compared to recombinant VLPs), and no neutralization activity, even at high concentration, in a pseudovirion neutralizing assay [27,34].

### Changes in epitopes and antibody footprints by surface Plasmon resonance (SPR)

**Figure 4:**
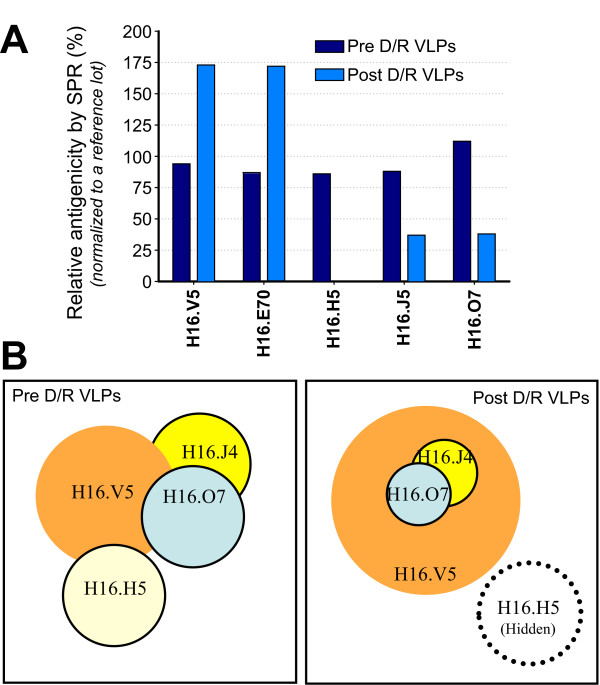
**Quantitative assessment of the impact of D/R on the binding affinities of different antibodies, specifically captured by chemically immobilized RAMFc **[**36**], **to HPV16 VLPs by SPR**. (**A**) Enhanced binding of neutralizing mAbs (V5, E70) and greatly reduced binding of linear epitope targeting mAbs (J4, O7 and H5) upon VLP reassembly. (**B**) Relative footprint changes of anti-HPV 16 L1 mAbs and their approximate degree of overlapping in pre- and post-D/R samples-data obtained with pair-wise epitope mapping.

To complement the IC50 ELISA analyses of antibody reactivity against HPV VLPs, we directly measured the antibody binding affinities for pre- and post-D/R VLPs SPR (Figure 4) by analyzing one mAb at a time without a need of a label or secondary detection reagents [35]. Binding signals are directly related to the mass deposited to the sensor chip for a given VLP:IgG binding pair. Consistent with the ELISA data, the SPR binding affinities for H16.V5 and H16.E70 were enhanced upon D/R, while the SPR binding affinities or their footprints for H16.H5, H16.J4 and H16.O7 were significantly reduced or abolished (in the case of H16.H5). A pair wise mapping study delineated the approximate footprint size and relative overlaps of the four mAbs (Figure 4).

### Visualization of VLPs and their binding to H16.V5 by cryoEM

**Figure 5:**
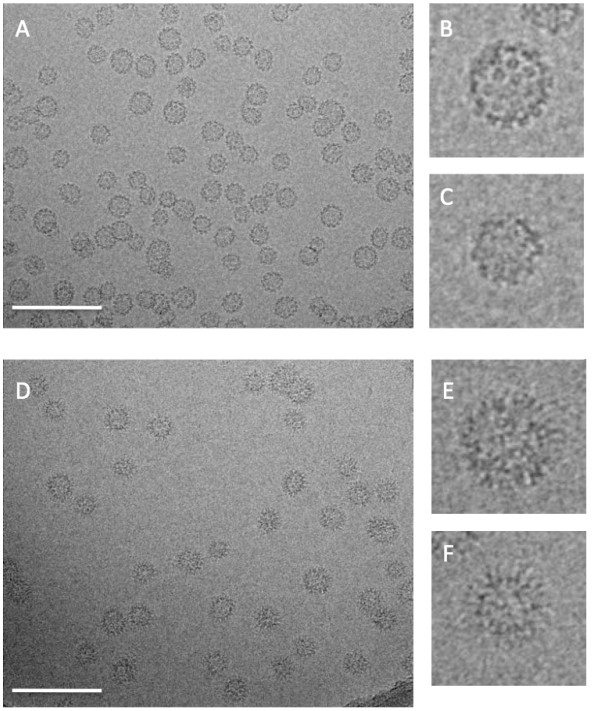
**(**A**) CryoEM image of HPV 16 VLPs post-D/R treatment shows a field of well-formed closed spherical particles**. (**B**,**C**) Larger scale images of individual VLP particles show that the individual capsomeres are arranged with a high degree of order. (**D**) CryoTEM image of post D/R HPV 16 VLPs mixed with H16.V5 Fab shows a similar field of particles densely decorated with antibody fragment. (**E**, **F**) Larger scale images of individual particles show that the antibody attachment is independent of the diameter of the individual particles. Scale bar is 200 nm.

The structure and morphology of various types of HPV VLPs before and after D/R have been compared directly by negatively stained transmission EM and atomic force microscopy (AFM) in previous studies [12,16,37]. To visualize the VLPs in a more native aqueous environment, we visualized post-D/R HPV VLPs by electron cryomicroscopy (cryoEM). An image of the sample (Figure 5A) shows that in addition to particles with the expected 55 nm diameter a substantial fraction of the particles have a smaller diameter of 40-44 nm and a few particles with a 20-nm diameter are also visible. Closer inspection of the particles (Figure 5B, C) shows that despite variations in the size and local curvature of the post-D/R VLPs, the particles are closed and fully assembled. Images of the HPV VLPs mixed with H16.V5 Fab show that all particles are densely decorated by the antibody regardless of their size (Figure 5D-F). However, we cannot rule out that other antibodies may be sensitive to structural differences between the 40- and 55-nm particles. When whole IgG of V5 was used, aggregates of VLPs were observed.

**Figure 6:**
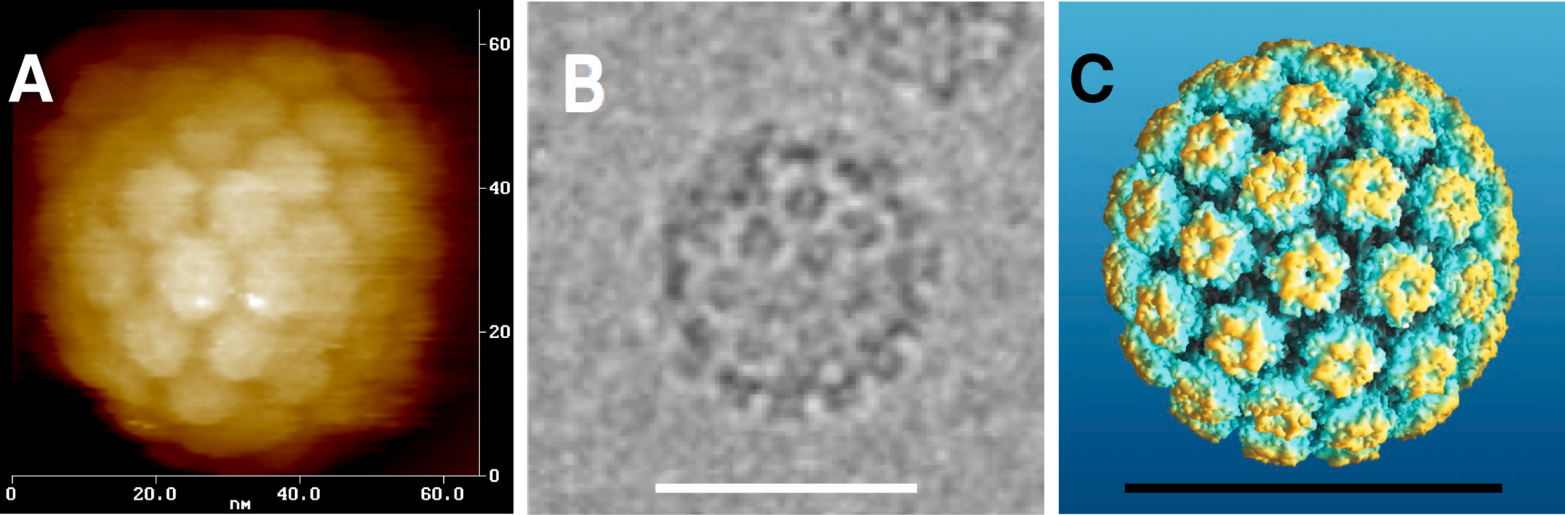
**HPV16 VLP dimensions and morphology**. (**A**) Atomic force microscopy image of a post-D/R HPV16 VLP, adapted from [16]. (**B**) CryoEM image of a post-D/R HPV16 VLP. The scale bar is 50 nm long. (**C**) Atomic model of the T = 7 HPV16 VLP. The scale bar is 50 nm long. The model was generated as described in the Methods.

**Figure 7:**
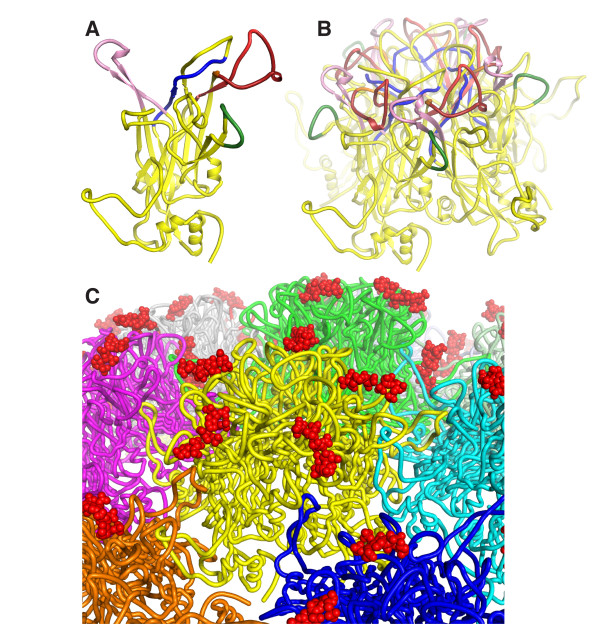
**Atomic model of HPV16 L1 in the context of the T = 7 VLP**. (**A**) A single subunit of L1 in the standard orientation. Residues 261-297 are in red and form part of the H16.V5, H16.E70, H263.A2 and H16.J4 epitopes. Residues 339-365 are in pink and form part of the H263.A2, H16.V5 and H16.E70 epitopes. Residues 174-185 are in dark green and form the H16.H5 epitope. Residues 111-130 are in blue and form the H16.I23 epitope. (**B**) A pentameric L1 capsomere with the same coloring scheme as in *(A) *and with the subunit in the foreground in approximately the same orientation as in *(A)*. (**C**) A view of the HPV16 VLP surface with the capsomere in yellow in the same orientation as in *(B) *and heparin oligosaccharides as they bind to L1 capsomeres shown in red. The atomic model was generated as described in the Methods.

A significant fraction of the particles in the cryoEM images have a diameter and capsomere arrangement that is consistent with the atomic force microscopy (AFM) data (Figure 6A, B). Moreover, the ~55 nm diameter and capsomere arrangement, as visualized by single particle image (AFM and cryoEM) are consistent with the atomic homology model of HPV16 VLP that we generated here (Figure 6C). The atomic homology model of the HPV16 VLP was constructed by superimposing the crystal structure of the HPV16 L1 pentamer [38] onto the core pentamer in the asymmetric unit of the high-resolution cryoEM structure of bovine papillomavirus type [39]. A single HPV16 L1 chain of was then superimposed on the sixth chain of the asymmetric unit of the BPV1 structure. HPV16 residues 2-21, 82-95, 404-437 and 475-488 were modeled based on the homologous BPV1 residues in the BPV1 cryoEM. The atomic model of the full T = 7 HPV16 VLP (Figure 7C) was generated by applying standard icosahedral symmetry operators to the asymmetric unit (see Methods for additional details).

### Epitope mapping based on the antibody binding affinities to post-D/R VLPs using an atomic model of HPV16

**Figure 8:**
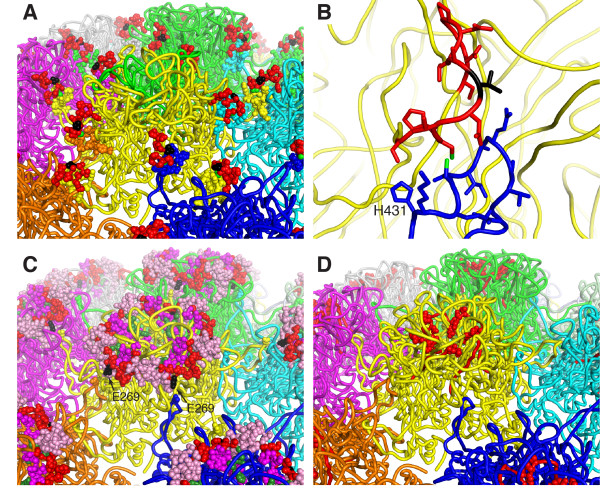
**Fine mapping of antibody epitopes on the HPV16 capsid**. (**A**) Same view as in Figure 7B, with the H16.H5 epitope (residues 173-185) shown in red. Side chains in the C-terminal arms of neighboring capsomeres that partially occluding the H16.H5 epitope are shown as colored spheres (not red). The conserved disulfide bond between Cys175 and Cys428 is highlighted with green spheres. The HPV16-specific Val178 is shown in black. (**B**) Close-up of the H16.H5 epitope from the subunit in the foreground of *(A) *with the same coloring scheme as in *(A)*. (**C**) Same view as in *(A)*, with the H16.V5, H16.E70, H263.A2 and H16.J4 epitopes highlighted: residues 261-265 (H16.J4 epitope only) are in dark green, residues 266-280 (in all four antibody epitopes) are in red, residues 281-297 (not part of the H16.J4 epitope) are in magenta. Residues 339-365 are in pink (not part of the H16.J4 epitope). Glu269 is shown in black. (**D**) Same view as in *(A)*, with the H16.I23 epitope (residues 111-130) in red.

To understand why the epitopes of certain antibodies become more exposed upon D/R treatment of VLPs while other epitopes become less exposed, we generated an atomic model of the entire HPV16 (T = 7)VLP (Figure 6) by fitting the crystal structure of HPV16 L1 [38] onto the 3.6 Å resolution cryoEM map of bovine papillomavirus type 1 (BPV1) [39]. The epitopes of the antibodies studied here have been coarsely mapped by measuring antibody reactivity against overlapping synthetic linear peptides (see Table 1 for references). The H16.H5 and H16.O7 epitopes were mapped in this manner to residues 174-185, in the EF loop [23]. While H16.H5 showed significant binding on the purified VLPs from yeast, H16.H5 did not bind post-D/R VLPs, and D/R treatment significantly reduces H16.O7 binding (Figures 3, 4). Residues 184 and 185 are not solvent-accessible in the pentameric L1 capsomere and are therefore unlikely to be part of any antibody epitope (Figure 7B). Residues 174, 176 and 179-183 are fully exposed in the VLPs (Figure 8A, B), implying that these residues are not sufficient for H16.H5 binding or for optimal H16.O7 binding. Residues 175, 177 and 178 are occluded by the C-terminal arm of an adjacent capsomere in the HPV16 VLP (Figure 8A, B) but not in pentameric capsomeres (Figure 7B). Moreover, we note that in our atomic model Ser173 forms a hydrogen bond with His431 from the C-terminal arm of an adjacent capsomere (Figure 8B). This interaction results in the occlusion of Ser173 upon VLP assembly. We therefore conclude that the occlusion of residues 173, 175, 177 and/or 178 upon completion of VLP assembly with all capsomeres in place is responsible for the loss in binding to H16.H5 and H16.O7, with the HPV16-specific Val178 playing a key role (see Discussion).

H16.J4 is a weakly neutralizing antibody originally identified by the immunoreactivity of the sera from HPV infected patients with cervical cancer [17,30]. Like H16.O7, H16.J4 bound significantly more weakly to fully assembled post-D/R VLPs than to pre-D/R VLPs (Figures 3, 4). The H16.J4 epitope has been mapped to residues 261-280 [17,25], in the first part of the FG loop, which partially overlaps with the epitopes of H16.V5, H16.E70 and H263.A2. The epitope is located in a loop on the crown of the L1 capsomere (Figure 7B) and is conserved across several types. Most of the residues are exposed on the viral surface in the HPV16 VLP atomic model, with the exception of residues 261-264 and 275-277. These residues form packing interactions that anchor the loop onto the core of the capsomere. Glu269 is partially occluded by Arg365, with which it forms a salt bridge. Additionally, accessibility of Glu269 is restricted by a number of other neighboring side chains including that of Gln424 from the C-terminal arm of an adjacent capsomere (Figure 8C). We conclude that the partial occlusion of Glu269 upon completion of VLP assembly may be responsible for the decrease in binding to H16.J4, and that the epitope may also include residues 265-274 and 278-280 (see Discussion).

In contrast to H16.H5, H16.O7 and H16.J4, antibodies H16.V5, H16.E70 and H263.A2 are neutralizing and their binding to VLPs was enhanced upon D/R treatment (Figures 3, 4). The epitopes of these three antibodies are contained within two surface loops on the crown of the L1 capsomere, residues 266-297 in the FG loop and residues 339-365 in the HI loop (Table 1, Figures 7B, 8C). Of these, the only residue that may be occluded upon completion of VLP assembly is Glu269, as noted above. Since binding was enhanced upon D/R, Glu269 is unlikely to play an important role in binding to H16.V5, H16.E70 or H263.A2. Residues 289-297, 339-345, 355, 360, 362 and 364-365 can also be excluded from the epitopes because these residues are not solvent-exposed in the HPV16 L1 pentameric capsomere (or in VLPs). Residues 286-288, 357 and 363 are also largely buried. We conclude that residues 266-268, 270-285, 346-354, 356-359, 362 and 363 form the core of the epitopes of H16.V5, H16.E70 and H263.A2 on the apex of a capsomere (Figures 7, 8C).

Our observations on H16.H5 and other linear targeting mAbs are consistent with the loss in binding when (pre-D/R) baculovirus derived HPV16 L1VLP-coated plates are compared to HPV16 L1 + L2 pseudovirion-coated plates [27]. Another unique mAb, H16.I23, recognizing residues 111-130 (in strand D and the DE loop), exhibited the same behavior: total loss of binding to pseudovirions. Within this epitope only residues 126-128 and 130 are exposed in the HPV16 atomic model. These residues are also exposed in unassembled capsomeres, and none of the buried residues are occluded by intercapsomeric interactions associated with virus assembly (Figure 8D).

## Discussion

Biophysical data from AFM, cryoEM, dynamic light scattering and sedimentation experiments show a significant improvement in the morphology, homogeneity and thermal stability of the HPV16 VLPs upon D/R treatment [12,37]. Antigenicity, which can be measured in vitro, is an accurate and convenient metric for product quality and stability, as a surrogate marker for in vivo immunogenicity of the VLP. For quantitative antigenicity analysis, sandwich ELISA has high specificity and sensitivity and can be developed for VLP characterization and product release with the desired mAbs. However, for bioprocessing or stability studies of final vaccine products, the interpretation of sandwich ELISA data can be complicated as both the capture Ab and detection Ab contribute to the final assay signals. Therefore, to understand the impact of D/R on VLPs for different epitopes, a set of fluorescence-based competitive ELISA assays with high specificity and sensitivity, allowing VLPs to freely interact with a given mAb in solution, was developed for probing epitope specific antigenicity on VLPs [28,29]. The quantitative ELISAs with half-maximal inhibitory concentration (IC50), by putting mAbs one at a time in the assay, yielded quantitative information on the impact of D/R treatment on individual epitopes.

Among an array of immunochemical assays for HPV VLP epitope characterizations, including competition ELISAs (IC50), sandwich ELISA, equilibrium dissociation constant determination, relative antigenicity and pair-wise epitope mapping, the epitope specific relative IC50, or rIC50, assay is the most sensitive assay with straightforward data interpretation due to the use of a single mAb in the assay. A ~3-fold enhancement in IC50 was observed for H16.V5 after D/R treatment of VLPs. In the relative antigenicity assay by SPR, the enhancement was ~2-fold. In addition, in a sandwich ELISA in which VLPs were captured with H16.J4 and detected with H16.V5, a moderate 30%-50% increase was observed [19,20]. This is not surprising given that these two mAbs showed opposing effects for binding to the post D/R VLPs. The sandwich assay implies a more dominant contribution from the detection H16.V5 since an overall increase of antigen content was seen, and conversely a smaller contribution by H16.J4 due to poorer capture of post-D/R VLPs (Figure 3B). Efficient D/R was shown to yield more fully assembled and presumably more virion-like VLPs with greatly reduced binding to H16.J4 and H16.O7, while rendering H16.H5 binding completely undetectable. This is consistent with a study by Culp et al. [27] in which pseudovirion binding was essentially undetectable for H16.H5, H16.J4, H16.O7 and H16.I23 while binding of each of these antibodies to L1-only VLPs derived from insect cells was readily detectable. It is thus conceivable that the epitopes for H16.H5 and H16.I23 were initially exposed in the imperfectly assembled VLPs after purification (from yeast or insect cells), and subsequently became inaccessible or buried after the formation of virion-like seamless VLPs after D/R during bioprocess or in the L1 + L2 pseudovirions. Since the H16.I23 epitope is not close to any intercapsomeric interfaces, the loss of H16.I23 binding to post-D/R VLPs and pseudovirions may be due to the rigidification of loops on the capsomere surface that may accompany particle assembly thereby restricting accessibility to the epitope. Our observations also caution against interpreting data from the widely used sandwich ELISA, particularly with respect to the dis- and reassembly of VLPs, where opposing and convoluted effects could be seen on the capture Ab and detection Ab.

The events that are likely to occur during D/R treatment on purified VLPs are: (1) breakdown of non-specific aggregates including disulfide-bonded aggregates (an overall reduction in particle size and increase in monodispersity were observed by dynamic light scattering and analytical ultracentrifugation); (2) unmasking of some virion-like epitopes previously hidden due to non-covalent or covalent association with other VLPs or capsomeres through aggregation or non-native disulfide bond formation; (3) promotion of complete assembly of the closed icosahedral VLPs; (4) reduction of non-native disulfide bonds and formation of native and thermodynamically preferred intra- and intercapsomeric disulfide bonds; (5) formation of native electrostatic and hydrophobic interactions upon correct capsomere assembly by formation of intercapsomeric disulfides [40,41]. Making VLPs with epitopes resembling those of authentic virions is essential in vaccine production. Understanding and quantitating the epitopes with multiple non-overlapping mAbs on the recombinant VLPs may provide insights on the understanding of not only serotype specific protection for vaccine types, but cross protection of non-vaccine types, by current vaccines [42].

Mapping of the previously identified antibody epitopes onto the HPV16 VLP atomic model shows that intercapsomere contacts in the VLP can explain the observed changes in antibody reactivity upon D/R and allow more precise and more consistent mapping of the antibody epitopes. Within the H16.H5 epitope (residues 174-185), residues 175, 177 and 178 are occluded by the C-terminal arm of an adjacent capsomere in the HPV16 VLP but not in pentameric capsomeres, suggesting that the observed reduction of H16.H5 and H16.O7 antibody reactivity to VLPs upon D/R is due to the occlusion of one or more of these residues. Cys175 forms an intercapsomeric disulfide with Cys428 in the C-terminal arm of a neighboring capsomere [39,43]. When the VLPs are fully assembled during D/R treatment, solvent access to the EF loop bearing Cys175 is thus occluded. VLPs that bind to H16.H5 antibody are therefore likely to have incomplete intercapsomeric disulfide formation, to be missing some capsomeres, or to be distorted such that H16.H5 can still access the epitope. However, residues 175 and 177 are widely conserved across HPV strains. Since the observed reduction of H16.H5 and H16.O7 binding to VLPs upon D/R is limited to HPV16, the reduction in binding cannot be fully explained by the occlusion of residues 175 and 177. Residue 178, which is a valine only in HPV16, is the only unconserved residue to be occluded upon VLP assembly. We therefore propose that Val178 plays a key role in H16.H5 and H16.O7 binding to misassembled (pre-D/R) VLPs or to unassembled HPV16 L1 in GST-L1 fusion protein [44] or in individual capsomeres, and that the occlusion of Val178 upon correct VLP assembly is largely responsible for the loss of reactivity against these antibodies. Moreover, we note that in our atomic model Ser173 forms a hydrogen bond with His431 from the C-terminal arm of an adjacent capsomere (Figure 8A, B). This interaction results in the occlusion of Ser173 upon VLP assembly. Since the Ser173-His431 pair is present only in HPV16, Ser173 may also be part of the H16.H5 and H16.O7 epitopes, even though Ser173 lies just outside the previously identified linear epitope of H16.H5 and H16.O7. The occlusion of Ser173 upon VLP assembly would provide an additional explanation for the reduction in antibody reactivity. We note that epitopes that are near interacapsomeric interfaces, such as those of H16.H5 and H16.O7, are more likely to exhibit antigenic differences between the 40-nm and 55-nm particles since structural differences between the two types of particles are likely to be concentrated at the interfaces between capsomeres. However, there is no evidence of any antigenic differences between the two types of particles.

In contrast to H16.H5, VLP binding to antibodies H16.V5, H16.E70 and H263.A2 is enhanced upon D/R treatment. The 3.6-Å resolution cryoEM structure of BPV1 and our atomic model of HPV16 do not, however, suggest that any epitopes become exposed upon virus assembly [39]. We conclude that the enhanced binding to post-D/R VLPs by H16.V5, H16.E70 and H263.A2 is most likely due to the decrease in aggregation in post-D/R samples, which was observed by dynamic light scattering with much smaller polydispersity indices (unpublished data) and by AFM and EM images [12,16]. We note that the epitope of these three antibodies contains an unpaired cysteine, Cys345, which is located within a few Ångström of the outer surface of the capsomere. Cys345 may be exposed to solvent in misfolded or improperly assembled VLPs, which could cause VLP aggregation through the formation of non-native disulfides.

## Conclusions

HPV16 L1 VLPs in Gardasil^® ^have undergone D/R treatment to produce more homogeneous VLPs with improved neutralizing epitopes, structure and stability. The effects on VLPs due to D/R treatment are evident in improving particle uniformity and size distribution from AFM and cryoEM images. Markedly different antigenicity of HPV16 VLPs was observed upon D/R treatment with a panel of monoclonal antibodies targeting neutralization sensitive epitopes. For VLP-based vaccine design and production, generating the virion-like epitopes consistently, keeping them stable and knowing how to analyze them accurately are of paramount importance. These mAbs targeting different epitopes function as the indicators for the consistency and correctness of VLP assembly. Significant binding to surface or buried linear epitopes (such as H16.J4, H16.O7 and H16.H5) is an indication of poor folding and/or incomplete assembly, as explained by the atomic model in this report. A similar approach, using the immunoreactivity of a mAb recognizing a linear epitope, was employed to probe the "incorrectly" or poorly folded erythropoietin [45,46]. Multiple epitope-specific assays with a panel of mAbs with different properties and epitopes are required to gain a better understanding of the immunochemical properties of VLPs and to correlate the observed changes at the molecular level. This type of insight into the variations in the VLP surface structure could facilitate the process development and finalization of desirable VLP product attributes and the formulation composition for further pre-clinical and clinical vaccine development.

## Methods

### HPV 16 L1 VLPs

Full length HPV16 L1 protein was over expressed in yeast. Purification of the VLPs, and the D/R process at lab scale was previously described [12,40] and the full scale production was performed using similar procedures. The protein concentration was determined with a BCA method.

### Anti-HPV 16 L1 mouse mAbs (IgGs) and fab of H16.V5

Mouse mAbs [17,18,27] were produced in cell culture or ascites using the cell lines provided by Prof. Christensen (Pennsylvania State University). Standard Protein A chromatography was used to purify IgGs. The concentration was determined with UV absorbance at 280 nm. Purity of the IgGs (> 95% pure) was determined with size exclusion HPLC and the isotype was verified with IsoStrip (Roche).

In order to prevent cross-linking of the VLPs during cryoEM experiments (with both IgG and Fab), Fab antibody fragments were prepared from the H16.V5 (IgG2b). Reduction of disulfide bonds and specific cleavage at the hinge region were achieved by adding a solution of H16.V5 (1.0 mg/ml) in 20 mM L-cysteine hydrochloride monohydrate to immobilized papain on agarose beads (Pierce), followed by gentle mixing for complete digestion over the course of two days at ambient temperature. Supernatant containing the Fab fragments was purified via gentle mixing with immobilized Protein A (Repligen) for one hour. Fab fragments in solution were concentrated using a 10 kDa filter (Centriprep YM-10, Millipore). The completeness of the cleavage of IgG and purified Fab was assessed with a size-exclusion HPLC. Concentrations of the H16.V5 Fab fragments were determined before and after filtration by UV absorbance measurements at 280 nm using an Agilent 8453 UV-Visible spectrophotometer.

### Assessment of epitope specific antigenicity by competitive fluorescence ELISA

Competitive fluorescence ELISA (FL-ELISA) of HPV VLPs was employed to assess the median inhibitory concentrations (IC_50_) of antigen in solution with half- maximal binding. Briefly, a fixed amount of HPV 16 VLPs on plate and varying amount of HPV L1 VLPs in solution were allowed to compete for the binding to a constant concentration of anti-HPV 16 mAbs at 10 or 20 ng/mL. This assay was used to quantitatively measure the solution antigenicity of HPV L1 VLPs against a given mAb. Reagent VLPs were passively adsorbed to a high-binding microtiter plate at a fixed concentration (e.g. 5 μg/mL of L1 protein and 100 μL per well for plate coating, pre- or post-D/R VLPs). 1.5-fold serial dilutions of a reference lot and the test samples were performed on the VLP coated assay plate using a Beckmann Biomek^® ^2000 or 3000 Laboratory Automation Workstation. A constant amount of the anti-HPV 16 antibody was then added to the solution HPV 16 VLP dilution series and the plate was incubated for 60 min. After washing away components in solution (free antibody, free VLPs, and VLP-antibody complex in solution), the antibody bound to the surface immobilized VLPs was quantitated with an alkaline phosphatase-conjugated rabbit anti-mouse IgG antibody. For detection, 4-methylumbelliferyl phosphate (4-MUP) was used as a substrate. The substrate forms the fluorescent substance 4-methylumbelliferone (4-MU) in the presence of the immobilized alkaline phosphatases. This fluorescence intensity was measured with a fluorescence plate reader after 60-70 min fluorescence development. Percentage of inhibition was calculated based on the wells without any antigen present in solution as a control, obtained on the same plate. Curve-fitting and IC50 calculation were performed with GraFit [47].

### Quantitative assessment of impact on different epitopes upon VLP reassembly by surface Plasmon resonance (SPR)

Epitope specific antigenicity assessments using SPR with a panel of mAbs were carried out using similar procedures as described [36]. Briefly, using a Biacore 3000 instrument, CM5 sensor chips were prepared by covalently coupling rabbit-anti-mouse IgG Fcγ antibodies (RAMFcγ) through the carboxylate groups in the dextran matrix on the sensor chip and the amine groups on the RAMFcγ using a Biacore Coupling Kit. The immobilized RAMFcγ antibodies on the chip surface were used to capture anti-HPV 16 L1 mAbs. HPV VLP samples (10 μg/ml) were injected onto the chip surface with specific mAbs captured. The extent of VLP binding to the anti-HPV 16 L1 mAb was measured as the mass increase at the sensor chip. The RU ratio was tracked for the VLP binding (RU_VLP_) to a given amount of captured IgG (RU_IgG_). Results (i.e., the ratios of RU_VLP_/RU_IgG_) from the samples are compared to results from a reference lot tested in adjacent cycles.

For pair-wise epitope mapping, VLP was first captured by H16.J4 (chemically immobilized) onto the sensor chip surface. Then the VLP surface was saturated with mAb1 by injecting mAb1 in high concentration (200 μg/mL), then the remaining VLP surface was probed by the binding of mAb2 to compare the degree of overlapping or the decrease in mAb2 binding to the mAb1-saturated VLP surface as compared to mAb2 binding to the free VLP. Then, in a separate assay cycle, the order of binding (mAb1:mAb2) was reversed, by saturating VLP with mAb2 first, followed by binding of mAb1.

### CryoEM of HPV 16 VLPs in hydrated form

Samples were preserved in a thin layer of vitrified ice over C-Flat holey carbon films (Protochips, Inc.) supported on 400 mesh copper grids. Samples were not diluted prior to vitrification. Electron microscopy was performed using a Tecnai F20 electron microscope (FEI Co.) operating at 120 keV equipped with a Gatan 4 k × 4 k digital camera. Images were acquired, using the Leginon data collection software [48], at a nominal magnification of 50,000x, corresponding to 0.226 nm/pixel at the specimen, using a dose of ~16 e^-^/Å^2^, and a nominal defocus of ~3 μm. The images of the V5-Fab with VLP were obtained by mixing them at 1:1 ratio based on protein concentration, prior to applying to the grid.

### Atomic model of T = 7 icosahedral HPV16 VLPs

The atomic homology model of the HPV16 VLP was constructed as follows. The crystal structure of the HPV16 L1 pentamer bound to heparin oligosaccharides (PDB code 3OAE) [38] was superimposed onto the core pentamer in the asymmetric unit of the high-resolution cryoEM structure of the bovine papillomavirus type 1 (BPV1) outer capsid (PDB code 3IYJ) [39]. A single L1 chain of HPV16 was then superimposed on the sixth chain of the asymmetric unit of the BPV1 structure. HPV16 residues 2-21, 82-95, 404-437 and 475-488 were absent or incompatible with particle assembly in the crystal structure. These residues were therefore modeled based on the homologous BPV1 residues in the BPV1 cryoEM structure using COOT [49]. The energy and geometry of the resulting atomic model of the full length HPV16 L1 VLP asymmetric unit were minimized with REFMAC [50]. The atomic model of the full T = 7 HPV16 VLP was generated by applying standard icosahedral symmetry operators to the asymmetric unit.

## Abbreviations

AFM: Atomic force microscopy; BPV: Bovine papillomavirus; cryoEM: Electron cryomicroscopy; D/R: Disassembly/reassembly; HPV: Human papillomavirus; IC50: Half-maximal inhibitory concentration; mAb: Monoclonal antibody; SPR: Surface plasmon resonance; VLP: Virus-like particle.

## Competing interests

The authors declare that they have no competing interests.

## Authors' contributions

QZ, KH, VT, Yuan Meng, YW, JA, MK, and MWW disassembled and reassembled the virus-like particles and carried out the ELISA and surface plasmon resonance experiments. DA, DW and MK generated the virus-like particles. Yorgo Modis carried out the molecular modeling studies, wrote the corresponding sections of the manuscript, edited the final manuscript and figures and handled the submission and peer review process. BC and CSP carried out the electron microscopy. QZ, MWW and RDS conceived of the study, and participated in its design and coordination and helped to draft the manuscript. All authors read and approved the final manuscript.
